# Women's attitudes towards mechanisms of action of family planning methods: survey in primary health centres in Pamplona, Spain

**DOI:** 10.1186/1472-6874-7-10

**Published:** 2007-06-27

**Authors:** Jokin de Irala, Cristina Lopez del Burgo, Carmen M Lopez de Fez, Jorge Arredondo, Rafael T Mikolajczyk, Joseph B Stanford

**Affiliations:** 1 Department of Preventive Medicine and Public Health, School of Medicine, University of Navarra, Irunlarrea 1, 31008 Pamplona, Spain; 2 School of Public Health, University of Bielefeld, Germany; 3 Department of Family and Preventive Medicine, School of Medicine, University of Utah. Salt Lake City, UT, USA

## Abstract

**Background:**

Informed consent in family planning includes knowledge of mechanism of action. Some methods of family planning occasionally work after fertilization. Knowing about postfertilization effects may be important to some women before choosing a certain family planning method. The objective of this survey is to explore women's attitudes towards postfertilization effects of family planning methods, and beliefs and characteristics possibly associated with those attitudes.

**Methods:**

Cross-sectional survey in a sample of 755 potentially fertile women, aged 18–49, from Primary Care Health Centres in Pamplona, Spain. Participants were given a 30-item, self-administered, anonymous questionnaire about family planning methods and medical and surgical abortion. Logistic regression was used to identify variables associated with women's attitudes towards postfertilization effects.

**Results:**

The response rate was 80%. The majority of women were married, held an academic degree and had no children. Forty percent of women would not consider using a method that may work after fertilization but before implantation and 57% would not consider using one that may work after implantation. While 35.3% of the sample would stop using a method if they learned that it sometimes works after fertilization, this percentage increased to 56.3% when referring to a method that sometimes works after implantation. Women who believe that human life begins at fertilization and those who consider it is important to distinguish between natural and induced embryo loss were less likely to consider the use of a method with postfertilization effects.

**Conclusion:**

Information about potential postfertilization effects of family planning methods may influence women's acceptance and choice of a particular family planning method. Additional studies in other populations are necessary to evaluate whether these beliefs are important to those populations.

## Background

To ensure women's right to a free choice in family planning (FP), the World Health Organization (WHO) recommends that information related to FP should include, at a minimum, the following for each method: effectiveness, correct use, mechanism of action, side-effects, health risks and benefits, reversibility, and protection against sexually transmitted infections [1]. Knowledge about each of these aspects can have practical implications regarding acceptance and satisfaction with the chosen method as well as minimize user errors. It is also important to acknowledge that women have different preferences and can accordingly make a choice of the method best suiting their wishes.

According to available evidence, some FP methods, including oral contraceptives, emergency contraception, and intrauterine devices, can act before and occasionally after fertilization [2-12]. Postfertilization effects may include structural and biochemical endometrial changes as well as alterations in fallopian tube motility. These effects may prevent implantation or pre-implantation embryonic development. The contribution of postfertilization effects to the overall effectiveness is potentially different for different methods [13]. Some authors have pointed out that postfertilization effects could be an important issue for some women, especially those who believe that human life begins at fertilization [14,15]. Apart from individual convictions, the preferences may differ by cultural background [14,16-18].

Mechanism of action was included in previous research in Europe regarding choice of FP methods in a general way [17,19-21], but there was no specific assessment of understanding and attitudes for postfertilization effects.

The purpose of our study was to assess the attitudes about the mechanisms of action of FP in terms of whether these may influence a woman's decision to choose an FP method or continue to use the chosen method. We also investigated which opinions and characteristics were associated with these decisions.

## Methods

We carried out a cross-sectional survey in a sample of women in reproductive age (18–49) from ten Primary Care Health Centres in various areas of Pamplona, Spain. Women attend this kind of centre for obtaining primary care from family physicians. These centres are part of the National Health Service and they do not have any particular religious affiliation. Prior to distributing the questionnaire women were asked about their age and women under 18 were excluded (age of majority in Spain). Additionally, the questionnaire contained a question about any surgery or known pre-existing condition causing infertility. Women who stated yes were asked to terminate the questionnaire just after initial questions and were excluded from the analysis.

We assumed that a fraction of respondents who will consider not using a family planning method sometimes acting after fertilization but before implantation will be in the range between 20 and 40% and we sought to estimate this proportion with ± 3% confidence interval, requiring a sample size of approximately 700 participants. We decided to include around 50–80 women per centre, depending on the population in each area; resulting in a total of 755 participants which is also a sufficient sample size for the multivariate analyses of the study [22].

An anonymous, self-administered, 30-item questionnaire about FP methods and medical and surgical abortion was administered to participants. The questionnaire was originally developed in English and translated into Spanish. One of the earlier English versions, containing all the relevant questions, was validated by assessing consistency of responses [15]. The translation was done by a bilingual (English-Spanish) speaker. A pilot study was carried out with 25 participants from a Primary Health Centre in order to identify any difficulties in understanding or completing the questionnaire. As a result of pilot testing, the wording of some questions was clarified. The centre where the pilot study was carried out was included among the study sites, but the data from the pilot project were not.

The following is an outline of the questionnaire: the first page was an informed consent form to participate in the study. The questionnaire then included a picture and an explanation of the female reproductive system and the human reproduction, stating: "In the next part of the questionnaire we ask some questions about how the different methods of birth control work. First, we will describe the stages of normal human reproduction:

"Stage 1": before fertilization-before the uniting of the sperm and the egg. *Birth control methods which do not allow sperm to get to the egg or that block ovulation itself are active at this stage*.

"Stage 2": after fertilization but before implantation-after the egg is fertilized but before it implants in the uterus; usually this takes 5–9 days from fertilization. During this time, the fertilized egg divides into an embryo of many cells. *Birth control methods which are active at this stage can sometimes stop development of the fertilized egg or embryo or destroy it*.

"Stage 3": after the embryo implants in the uterus. *Methods which are active at this stage can destroy the embryo*.

Subsequent sections were related to beliefs, attitudes, and personal preferences of FP method use according to what stage the mechanism of action of the method takes place. Women were asked about the most important three characteristics they take into account when choosing an FP method with an open-ended question. All attitude questions were related to a general hypothetical method of FP. In further sections women were asked about their knowledge of the mechanism of action of specific FP methods and if doctors or providers should explain the details of how a method works if it sometimes works after fertilization (stage 2) or after implantation (stage 3); the results were reported in another analysis [23]. In Table 1, we listed the specific wording of the key questionnaire items. The questionnaire did not provide any information about how different methods of FP work. In several questions, medical and surgical abortion were listed among birth control or family planning methods and the questionnaire did not provide any specific definition of either birth control or family planning. We are aware that medical and surgical abortion are not strictly considered as birth control methods, but they are often offered to prevent unwanted births [24,25]. Religiosity was measured by two variables: church attendance (How often do you attend church or worship services?, with a six points scale from "more than once a week" to "never") and personal importance of faith (How much do you agree with the following statement: "My faith is the most important influence in my life"?, with a five point scale from "strongly agree" to "strongly disagree"). Women who attended church once per week or more often and considered faith the most important influence in their life were classified into a "high religiosity" group, the remaining into "low religiosity". The low religiosity group included also women without religious affiliation. Demographic information was asked at the conclusion of the questionnaire. The Spanish and the English version of the questionnaire are available from the authors upon request.

**Table 1:** Key questions from the questionnaire *

8. In some cases there is a loss of a fertilized egg or an embryo because of natural causes at Stage 2 or 3. However the loss can be also caused by some birth control methods. Is it an important difference for you if the loss of an embryo is natural or caused by a method of birth control? (*Yes/No/Unsure*)
10. Would you consider using a birth control method that sometimes works after fertilization but before implantation (**Stage 2**)? (*Yes/No/Unsure*)
11. If you were using a birth control method, and you learned that it sometimes works after fertilization but before implantation, would you stop or continue using the method? (*Stop/Continue/Unsure*)
12. Does your above choice depend on how often the given method works after fertilization but before implantation? (*Yes/No/Unsure*)
If Yes – how often would the method have to work at Stage 2 to make you stop using the method? (*More than one time in one year/More than one time in ten years/More than one time in 100 years: if 100 women use the method one of them experience it in each year/Other (please, specify)/Do not know*)
13. Would you consider using a birth control method that sometimes works after implantation in the uterus (**Stage 3**)? (*Yes/No/Unsure*)
14. If you were using a birth control method, and you learned that it sometimes works after implantation in the uterus (Stage 3), would you stop or continue using the method? (*Stop/Continue/Unsure*)
15. Does your above choice depend on how often the given method works after implantation? (*Yes/No/Unsure*)
If Yes – how often would the method have to work at Stage 3 to make you stop using the method? (*More than one time in one year/More than one time in ten years/More than one time in 100 years: if 100 women use the method one of them experience it in each year/Other(please, specify)/Do not know*)
18. If you are using a birth control method that might sometimes work after fertilization but before implantation (**Stage 2**), should your doctor or provider tell you the details about how the method works? (*Yes/No/Unsure*)
19. If you are using a birth control method that might sometimes work after implantation (**Stage 3**), should your doctor or provider tell you the details about how the method works? (*Yes/No/Unsure*)
21. When do you believe human life begins? Please check the one that best applies. (*At some time before fertilization/At the time that the sperm and egg unite (fertilization)/At the time that the embryo implants into the uterus (implantation)/At the time that the embryo or fetus reaches a certain stage of development. What time or stage?/When fetus could survive on its own outside the uterus/At birth/Sometime after birth – when?/There is no exact time at which I can say that human life has definitely begun/I am not sure/I do not have an opinion/Other, please describe*)

* Possible answers of the questions are presented in brackets. The questions are numbered as in the questionnaire.

The questionnaire was distributed between March and May of 2004 by a female doctor and two female research assistants. There were no incentives for completing the questionnaire. Participation in the study was solicited at healthcare centres in Pamplona. As the questionnaire takes approximately 10–15 minutes to complete, women were able to do so while waiting for their doctor's appointment. Questionnaires were handed out and returned in a closed envelope to further ensure anonymity. The researcher team distributed the questionnaires in 2–3 consecutive days in each centre. Although it is not usual for a woman to visit the health care centre in consecutive days, the researchers asked each new participant if she had filled the questionnaire the day or days before to avoid repeated participation of same subjects.

Data were analysed with SPSS version 11.0 statistical software (SPSS Inc, Chicago, IL). We calculated proportions and their confidence intervals based on normal approximation. We used logistic regression to assess the characteristics independently associated with four outcome variables: (1) would use a method that occasionally works after fertilization (no versus yes or unsure), (2) would use a method that occasionally works after implantation (no versus yes or unsure), (3) would continue using a method after learning it works after fertilization (no versus yes or unsure) and (4) would continue using a method after learning it works after implantation (no versus yes or unsure). First, we included all variables that had a p-value <0.25 in the univariable logistic regression analyses in each of the multivariable models. The models were subsequently reduced by stepwise exclusion of variables which were not significant at the p-value <0.05 in the multivariable models [22].

We also performed an analysis of consistency in the responses, similar to what had been performed previously for an English version of this questionnaire [15]. We classified answers of women who stated they would not use methods acting between fertilization and implantation (stage 2) but that stated they would use methods acting after implantation (stage 3) as inconsistent.

Ethic Committee approval for the study was obtained at the University of Navarra. Permission to administer the questionnaire was obtained from the director of each of the centres involved in the study.

## Results

### Description of the sample

Seven hundred and fifty five participants were approached. Twenty eight women had any surgery or condition that makes a woman unable to get pregnant for the rest of the life or were over 49 years of age, and were excluded from the analysis. Twenty-nine women (4%) chose not to participate in the study, 14 of them were not interested and 15 women said that they did not have enough time. Forty-three of the eligible questionnaires (6%) were not returned and 74 (10%) did not contain enough information to be analyzed, giving a response rate of 80% and final sample size of 581.

Inconsistent responses according to stage 2 and 3 were found in 12.2% of the sample. All analyses excluding women with inconsistent responses were repeated yielding substantially the same results as presented below.

The respondents were mostly Spanish women with a mean age of 30.8 (SD = 7.01), and had annual incomes between 20 and 40,000 €. The majority had completed some form of post-high school education. Nearly half of the participants were married (47.8%). The majority of women were Catholic, but attended church occasionally (≤ 1 time/month) and did not consider faith to be an important influence in their life. Nine point seven percent of the participants were classified into the group with "high religiosity". There were no Muslim, Hindu or Buddhist participants. The majority of participants expressed that they would like to become pregnant at some time in the future. Most of the women had no children yet (Table 2). The most common methods of family planning ever used by participants were condoms (78%) and oral contraceptives (58%). The three most important characteristics to women in choosing a birth control method were: efficacy, convenience and easy use and absence of side effects. They were named by 76%, 53.4% and 28.6% of the surveyed women respectively. Other characteristics mentioned by some women were: beneficial health effects (like cycle control or protection against sexually transmitted infections) (7.4%), low cost (4.6%), easy access (2.4%), non abortive (2%), consistent with personal beliefs (1.2%), reversible (1%), acceptance by the partner (0.8%) and natural (0.8%).

**Table 2:** Characteristics of the participants

**CHARACTERISTICS**		**n (%)**	**CI 95%**
**Country of origin**	Spain	536 (92.4)	(90–94.4)
	Central/South-America	44 (7.6)	(5.6–10.1)
	Total	580 (100)	
**Education**	High school or less	116 (20)	(16.8–23.5)
	Technical college *	188 (32.4)	(28.6–36.4)
	University degree	241 (41.6)	(37.5–45.7)
	Doctorate (Ph. D.)	35 (6)	(4.2–8.3)
	Total	580 (100)	
**Annual income**	<20.000 €/year	163 (28.5)	(24.8–32.4)
	20–40.000 €/year	205 (35.8)	(31.9–39.9)
	>40.000 €/year	82 (14.3)	(11.6–17.5)
	Don't know	122 (21.3)	(18.0–24.9)
	Total	572 (100)	
**Marital status**	Married	276 (47.8)	(43.6–51.9)
	Single in committed relationship	157 (27)	(23.6–31)
	Single	129 (22.3)	(19.0–25.9)
	Other (separated, divorced, widow)	16 (2.8)	(1.6–4.5)
	Total	578 (100)	
**Religion**	None	171 (29.7)	(26.0–33.7)
	Catholic	401 (69.7)	(65.8–73.5)
	Other (Protestant, Evangelist)	3 (0.5)	(0.1–1.5)
	Total	575 (100)	
**Frequency of church attendance**^†^	Once a week or more	81 (20.6)	(16.6–24.9)
	Occasionally (≤ 1/mouth)	289 (73.4)	(68.7–77.7)
	Never	24 (6.1)	(3.9–8.9)
	Total	394 (100)	
**"Faith is the most important influence in my life" **^†^	Agree Disagree	120 (30.5) 146 (37.1)	(26.0–35.3) (32.3–42.0)
	Don't know	128 (32.5)	(27.9–37.4)
	Total	394 (100)	
**Desire for future pregnancy **^‡^	No	122 (21.1)	(17.9–24.7)
	Yes	455 (78.9)	(75.3–82.2)
	Total	577 (100)	
**Live births**	0	338 (58.2)	(54.1–62.2)
	1	121 (20.8)	(17.6–24.4)
	2	92 (15.8)	(12.9–19.1)
	>2	30 (5.2)	(3.5–7.3)
	Total	581 (100)	
**Elective abortions **^§^	0	260 (91.6)	(87.7–94.5)
	1	20 (7)	(4.4–10.7)
	2	4 (1.4)	(0.01–2.0)
	Total	284 (100)	
**Age**	18–24	117 (20.1)	(16.9–23.6)
	25–34	287 (49.4)	(45.3–53.5)
	35–44	160 (27.5)	(23.9–31.4)
	45–50	17 (3)	(1.8–4.6)

CI 95%: 95% confidence interval of the proportion.

* Technical college: a college offering students courses in technical and other subjects after they have left school.

^† ^Variables apply only to women who have a religious affiliation.

^‡ ^No: refers to women who clearly state that they do not want to get pregnant the future. Yes: refers to women who want to get pregnant in the future and those who are not sure about a future pregnancy.

^§ ^Variable refers only to women that have been pregnant in the past.

### Opinions and attitudes related to postfertilization effects

Approximately half of the participants (46.3%) believed that life begins at fertilization (Table 3). Most (58.7%) of the women stated that it is important for them to distinguish between natural embryo losses from those caused by family planning methods. The fraction was slightly higher (67.5%) among women who believed that life begins at fertilization.

**Table 3:** Women's opinions and attitudes related to postfertilization effects of family planning methods.

**OPINION OR ATTITUDE**		**n (%)**	**CI 95%**
**Human life beginning**	Fertilization	266 (46.3)	(42.1–50.4)
	Implantation	103 (18)	(14.8–21.3)
	After Implantation	102 (17.7)	(14.7–21.1)
	Other *	104 (18)	(15–21.4)
	Total	575 (100)	
**Embryonic loss' cause **^†^	Not important	141 (24.3)	(20.9–28.1)
	Important	33 (58.7)	(54.5–62.7)
	Unsure	98 (17)	(13.9–20.2)
	Total	578 (100)	
**Would consider using a method that**	Yes	222 (38.4)	(34.4–42.5)
**may work after fertilization**	No	228 (39.4)	(35.4–43.6)
	Unsure	128 (22.2)	(18.8–25.7)
	Total	578 (100)	
**Would consider using a method that**	Yes	84 (14.5)	(11.7–17.6)
**may work after implantation**	No	330 (57)	(52.8–61.1)
	Unsure	165 (28.5)	(24.8–32.4)
	Total	579 (100)	
**Decision about using a method**	Stop using	205 (35.3)	(31.4–39.3)
**after learning it may work after**	Continue using	183 (31.6)	(27.7–35.4)
**fertilization**	Unsure	192 (33.1)	(29.2–37)
	Total	581 (100)	
**Decision about using a method**	Stop using	325 (56.3)	(51.8–60)
**after learning it may work after**	Continue using	79 (13.7)	(10.9–16.7)
**implantation**	Unsure	173 (30)	(26.1–33.7)
	Total	581 (100)	

CI 95%: 95% confidence interval of the proportion.

* Other: includes the other options in the questionnaire: "there is no exact time", "I am not sure", "I do not have an opinion," and "sometime before fertilization".

^† ^Embryonic loss' cause: refers to whether it is important to distinguish natural embryo losses from those that may be caused by birth control methods.

Of all respondents, 39.4% reported that they would not consider using an FP method that sometimes work**s **after fertilization but before implantation (stage 2) and 57% would not consider using a method that sometimes works after implantation (stage 3). In addition, 35.3% of the women said they would stop using an FP method if they learned that it work**s **after fertilization but before implantation. This figure increased to 56.3% if the method in question works after implantation (Table 3). The questionnaire also asked whether the relative frequency of action at stage 2 or stage 3 would influence these attitudes. For 80.7% and 82.7% of the women it did not matter how often a method might work at stage 2 or stage 3 respectively.

Several variables were found to be independently related to a woman's decision to use a method that sometimes could work after fertilization but before implantation or stage 2 (Table 4). University graduates and women with a doctoral degree (Ph. D.) and those who believed that human life begins at implantation, at some point after implantation or are unsure about an exact moment, were more likely to report they would use a method that occasionally works after fertilization. In contrast, women with high religiosity are less likely to report they would use this kind of method (Table 4).

**Table 4:** Variables significantly associated with women's potential decisions about postfertilization effects of family planning methods

**VARIABLES ASSOCIATED WITH WOMEN'S DECISIONS**		WOMEN'S DECISIONS ODDS RATIO * (CI 95%)			
		
		**Would use a method that occasionally works after**		**Would continue using a method after learning it works after**	
		**fertilization**	**implantation**	**fertilization**	**implantation**
**Education**	High/technical college^†^	1 (ref.)		1 (ref.)	
	University graduate, doctorate degree (Ph.D.)	2.13 (1.45–3.13)		1.89 (1.25–2.86)	
**Human life beginning**	Fertilization	1 (ref.)	1 (ref.)	1 (ref.)	1 (ref.)
	Implantation	2.06 (1.22–3.50)	2.99 (1.35–6.62)	2.18 (1.20–3.95)	1.63 (0.68–3.94)
	After Implantation	4.24 (2.51–7.17)	6.24 (3.10–12.58)	8.18 (4.64–14.40)	6.41 (3.22–12.74)
	Other ^‡^	2.95 (1.75–4.96)	4.03 (1.93–8.40)	3.04 (1.73–5.36)	2.51 (1.17–5.38)
**Embryonic loss' cause**^§^	Not important	1 (ref.)	1 (ref.)	1 (ref.)	1 (ref.)
	Important	0.47 (0.32–0.69)	0.30 (0.18–0.52)	0.34 (0.22–0.51)	0.21 (0.12–0.38)
**Religiosity **^#^	Low	1 (ref.)		1 (ref.)	
	High	0.13 (0.04–0.43)		0.30 (0.22–0.51)	

* All logistic regression models are adjusted for the variables shown in the table and country of origin, annual income, marital status, age, desire for future pregnancy, number of pregnancies and number of elective abortions.

^† ^Technical college: a college offering students courses in technical and other subjects after they have left school.

^‡ ^Other: includes the other options in the questionnaire: "there is no exact time", "I am not sure", "I do not have an opinion", "sometime before fertilization."

^§^Embryonic loss' cause: refers to whether it is important to distinguish natural embryo losses from those that may be caused by birth control methods.

^#^High: women who strongly identify with a religion (e.g. attend church or worship services weekly and consider faith to be the most important influence in their life). Low: women with no religious affiliation or who identify with a religion but attend church or worship services occasionally (<1/mounth) and/or do not consider faith as the most important influence in their life.

Regarding hypothetical FP methods that sometimes work after implantation, women who believed that human life begins at implantation, at sometime after implantation or were unsure about the time when human life is beginning, were more likely to use them. However, those who believe that it is relevant to distinguish natural embryo losses from non-natural losses were less likely to use these methods (Table 4).

University graduates and women with a doctoral degree (Ph. D.) and those who believed that human life begins at implantation, at some point after implantation or were unsure about an exact moment that life begins stated they would be more likely to continue using an FP method that could work after fertilization, if they were to learn that their own FP method worked in this way. On the contrary, both those who believe that it is relevant to distinguish induced embryo loss from natural embryo loss and women with high religiosity affirmed they would be less likely to continue using these methods (Table 4).

Finally, women who believed that human life begins at some point after implantation or were unsure about the exact moment where life begins stated they would be more likely to continue using an FP method that could work after implantation, if they were to learn that their own FP method worked in this way. On the other hand, those women who considered that it is relevant to distinguish natural embryo losses from non-natural losses referred they would be less likely to continue using an FP method that occasionally works after implantation, if they were to learn that their own FP method worked in this way (Table 4).

Thus, both the beliefs about human life beginning and considering a difference between natural and other causes of embryonic loss were the sole variables independently associated with all four outcome variables.

## Discussion

We investigated the beliefs regarding postfertilization effects of FP methods in a convenience sample recruited in an urban area in northern Spain (Pamplona). Our results support the hypothesis that information about how FP methods work may affect women's decision-making process in choosing a method [2,26].

Our study shows that women who believe that human life begins at implantation or sometime after implantation are more likely to state that they would use an FP method with postfertilization effects in comparison to those who consider life to begin at fertilization. This conclusion is consistent with other published studies. Gould et al. found that after requesting specific information about the mechanism of action of emergency contraception, women who believed that human life begins at fertilization tended more to believe that emergency contraception was an abortive method because of its anti-implantation effect [26]. These results were also confirmed by Jackson et al [27]. Romo et al. found that a woman's religion was not associated with using emergency contraception. Rather, the mechanism of action was the major factor that differentiated women willing to use emergency contraception or not: those who believed that emergency contraception worked after fertilization were less willing to use it [14].

Our results show that religious beliefs can influence the use of methods with postfertilization effects. Spain is a predominantly Catholic country and the rejection of postfertilization effects is consistent with the Catholic understanding of the beginning of human life. This understanding is however not restricted to Catholicism and can also be found in other countries [28,29]. The independent importance of religion disappeared when methods working after implantation were considered. Perhaps there is a greater general consensus in society regarding the consideration that should be given to the human embryo after implantation as compared to after fertilization [9,30].

There are several limitations to our study. First of all, the surveyed women were not a systematically representative sample of the fertile female population of Spain or even Pamplona. However, this study was carried out in various areas of Pamplona, differing in socio-economic status, to take into account different socio-economic levels. In fact, our sample is quite similar to the Spanish female population in terms of demographic characteristics [31]. Also, although we explained in detail the physiology and the concept of stages used in this questionnaire with help of a picture, it is possible that some women had difficulty in understanding these concepts, which may have resulted in inconsistent responses. We found inconsistencies in 12.2% of the sample, indicating the difficulties in communicating the information about the mechanism of action. To assess the influence of inconsistent responses, we repeated all analyses excluding women with inconsistent responses, finding the same results as presented here.

We did not measure the choices and actual behaviours directly, but only the hypothetical choices: we asked whether women would consider using and would stop or continue using. In addition, hypothetical choices were examined in terms of a non-specific "method of family planning" and not in terms of actual methods such as oral contraceptive pills, etc. We do not know the extent to which these considerations would be followed in a real life decision making, involving specific FP methods, when different factors have to be weighted against each other. For example: how strong would women pursue their considerations regarding postfertilization effects if they were informed that other methods are less effective. In fact, efficacy, convenient/easy to use and no side effects were the three most important characteristics considered by the women when choosing an FP method. These results are consistent with other studies [32,33]. Other characteristics were referred by very few women. This could be due to the fact that women were queried using the open-ended question: "Which are the three most important features of family planning methods for you?" It is possible that women would have not thought of some specific characteristics in the moment they filled the questionnaire. For example, few women considered reversibility as an important factor, although the majority had used reversible methods. Also, only 2% spontaneously referred "being non abortive" as an important factor. But 57.3% of women who believed that human life begins at implantation, stated they will not be using a method that occasionally works after implantation (data not shown). More studies are needed to assess the importance of potential postfertilization effects in comparison with other characteristics of FP methods.

We also did not study men's preferences and beliefs. Although women may have the major role in choosing a method in developed societies, the decision process may include negotiation between both partners.

We endeavoured to make this questionnaire as neutral as possible in its wording, in order to avoid biasing responses by wording that would encourage women to respond either positively or negatively. In retrospect, we are aware that the question "*is it an important difference for you if the loss of an embryo is natural or caused by a method of birth control?*" could be interpreted in different ways. The term "natural" could be identified with "good", "acceptable" or "consistent with one's beliefs" and the term "caused by birth control method" with "bad", "unacceptable" or "inconsistent with one's beliefs". To our knowledge, this is the first study to tackle the issue of perception regarding natural losses in the context of postfertilization effects. Our results are not definitive and require further exploration. However, the issue is of importance as shown in the recent editorial on emergency contraception where the underlining ethical argument for the acceptance of this method was the minimization of any losses (regardless of their cause) [34,35]. Our interpretation at this point is that a substantial fraction of women consider the difference to be important and this is the reason why they may reject the use of methods which may cause losses. We found a confirmation for this in responses showing that this attitude rarely depended on the actual frequency of the caused losses.

In future research, we concur with the recommendation of a reviewer for a different wording of this question: *"Sometimes a fertilized ovum or an embryo does not continue to grow for a variety of reasons. Sometimes this happens on its own and sometimes medication, like a contraceptive method, may cause this to happen."*

We also did not ask the same questions about methods active at stage 1 as about those acting at stage 2 or 3. This might have bias the respondents in the way as to considering methods active at stage 2 or 3 as something bad. Previous research indicated moral considerations related to stage 2 or 3 are rather frequent, whereas anecdotal information points towards only rare reservations against methods working before fertilization [2,9]. We thus refrained from adding a question on whether the women would stop using an FP method acting at this stage. We do not know how much this omission could have affected the respondents but we believe it is unlikely to substantially explain our results.

Despite the limitations, our study has several strengths. We obtained a high response rate and very few women refused to participate in the study, so the possible volunteer bias is minimized. The implementation of the questionnaires was carried out in a short period of time in order to avoid the spread of information about the study among patients of the different health centres. As the questionnaire was self-administered, the interviewer bias was avoided. We did not include the term "abortifacient" when referring to the mechanism of action of some FP methods. We did not state the mechanism of action of any FP methods and thus asked women to express their beliefs independently of their actual choices and independently of their perceptions of available choices.

In our study, we also assessed women's understanding about the mechanisms of action of specific FP methods, and we also asked women if doctors or providers should explain the details of how a method works if it works after fertilization (stage 2) or after implantation (stage 3). A detailed description of those results has been published elsewhere [23]. Briefly, only a small minority of the surveyed women were aware that postfertilization effects may exist for oral contraceptives (4.7%), the emergency contraceptive pill (7%) and the intrauterine device (3.4%). Ninety one percent of the surveyed women referred that doctors or providers should inform them about the possibility of postfertilization effects.

The considerations not to use a method potentially acting after fertilization appear to be frequent in the population we studied. These decisions are not readily predicted from socio-demographic variables, but are rather associated with personal beliefs regarding the beginning of human life and the opinion that there is a moral difference between embryonic losses that occur spontaneously and those that may be caused by family planning methods. It may be difficult to assess such personal beliefs within the time constraints of the family planning clinics. On the other hand, a client is potentially done a disservice if the information provided conceals or downplays information about the mechanism of action that she finds relevant to her personal moral beliefs. Thus we believe that the information should be provided to all women rather than to subgroups with special characteristics. Alternative FP methods should be presented in a comprehensive way as otherwise women may feel uncomfortable compromising their moral concerns when alternatives do not appear available. If a woman is not fully comfortable with her chosen method of family planning, she may not use it as effectively.

## Conclusion

Our results emphasize that full information about the mechanism of action of FP methods is important for many women. Beliefs regarding action after fertilization were barely associated with socio-demographic or religious characteristics, but were strongly associated with personal beliefs surrounding the beginnings of human life. For those women who would not use or would stop using a method acting after fertilization, it did not matter whether such effects were common or rare. We believe that it is necessary to inform women about specific mechanisms of action of FP methods in order to provide an adequate and fully informed consent and to ensure women's right to a free choice. Future studies in other populations are necessary to evaluate the differences between different cultures in this respect.

## Competing interests

The author(s) declare that they have no competing interests.

## Authors' contributions

JI participated in the design and coordination of the study, supervised the statistical analysis and helped to draft the manuscript. CLB collected the data, performed the statistical analysis and drafted the manuscript. CMLF and JA also collected the data and performed the statistical analysis. JBS and RTM conceived the study, participated in its design and coordination and helped to draft the manuscript. All authors read and approved the final manuscript.

## Pre-publication history

The pre-publication history for this paper can be accessed here:


